# Improving Patient Non-Attendance Rates in a General Adult Community Mental Health Outpatient Clinic Using Telephone Reminder Phone Calls

**DOI:** 10.1192/bjo.2026.11470

**Published:** 2026-06-30

**Authors:** Shivani Richards, Oluwakemi Okopi

**Affiliations:** Leeds and York Partnership NHS Foundation Trust, Leeds, United Kingdom

## Abstract

**Aims:**

To assess whether reminder telephone calls improve patient ‘Did-Not Attend’ (DNA) rates; evaluate whether specific demographic groups are less likely to attend appointments.

Over 35% of psychiatric outpatient appointments are not attended. High DNA rates increase waiting times and lead to inefficient use of healthcare resources. They are associated with poorer health outcomes, medication non-adherence and higher chances of hospital re-admission.

Reasons for non-attendance are multifactorial, including socioeconomic factors, illness severity and forgetting appointments. Reminder telephone calls were utilised to assess their effectiveness in improving attendance.

**Methods:**

A retrospective review of patients booked for mental health outpatientappointments in a South-West Leeds community unit was conducted using electronic patient records (Caredirector). All patients had been sent appointment letters by post. Data was collected over a 16-week period and included 95 patients, of whom 22 patients DNA.

Reminder telephone calls followed a standardised script. Cycle 1 involved the reminder telephone call seven days before appointments, over a 4-week period (35 patients). Cycle 2 involved reminder telephone calls two days before appointments, over a 5-week period (21 patients). Two telephone call attempts were made if patients did not answer.

Qualitative data on appointment awareness and additional patient comments were recorded. Attendance and demographic data were then gathered from CareDirector.

**Results:**

In Cycle 1, 51.4% of patients answered the reminder telephone call, compared to 61.9% in Cycle 2. The average telephone call duration was 3 minutes 29 seconds. The baseline DNA rate prior to intervention was 23.16%, reducing to 14.3% with seven-day reminders and 19.1% with two-day reminders. However, amongst patients who confirmed attendance, subsequent attendance was higher with two-day reminders (100%) than seven-day reminders (83.3%). A higher DNA rate was observed amongst males in Cycle 1 (4:1), but rates were equal across genders in Cycle 2. No clear associations were identified between non-attendance and age, ethnicity, marital status, religion or interpreter requirement.

**Conclusion:**

Findings suggest that forgetting appointments contribute to non-attendance, as reminder telephone calls reduced DNA rates in community outpatient clinics.The intervention was time-efficient, supporting feasibility for routine clinical practice. This simple measure could improve patient outcomes and optimise the use of mental health service resources. Future cycles could assess text-message reminders for patients who do not answer reminder telephone calls, to see if this further reduces DNA rates.

